# Circulating Tumor Cell Count and Overall Survival in Patients With Metastatic Hormone-Sensitive Prostate Cancer

**DOI:** 10.1001/jamanetworkopen.2024.37871

**Published:** 2024-10-07

**Authors:** Amir Goldkorn, Catherine Tangen, Melissa Plets, Daniel Bsteh, Tong Xu, Jacek K. Pinski, Sue Ingles, Timothy Junius Triche, Gary R. MacVicar, Daniel A. Vaena, Anthony W. Crispino, David James McConkey, Primo N. Lara, Maha H. A. Hussain, David I. Quinn, Tanya B. Dorff, Seth Paul Lerner, Ian Thompson, Neeraj Agarwal

**Affiliations:** 1 Division of Medical Oncology, Department of Medicine, Keck School of Medicine of USC, Los Angeles, California; 2 SWOG Statistics and Data Management Center, Fred Hutchinson Cancer Center, Seattle, Washington; 3 USC Norris Comprehensive Cancer Center, Los Angeles, California; 4 Keck School of Medicine of USC, Los Angeles, California; 5 Children’s Hospital Los Angeles, University of Southern California, Los Angeles; 6 Illinois CancerCare PC, Peoria; 7 Holden Comprehensive Cancer Center, University of Iowa Health Care, Iowa City; 8 UsTOO Las Vegas, Las Vegas, Nevada; 9 Johns Hopkins Greenberg Bladder Cancer Institute, Baltimore, Maryland; 10 UC Davis Comprehensive Cancer Center, Sacramento, California; 11 Northwestern University Feinberg School of Medicine, Chicago, Illinois; 12 City of Hope Comprehensive Cancer Center, Duarte, California; 13 Scott Department of Urology, Dan L Duncan Cancer Center, Baylor College of Medicine, Houston, Texas; 14 Christus Health, San Antonio, Texas; 15 Huntsman Cancer Institute, University of Utah, Salt Lake City

## Abstract

**Importance:**

In metastatic hormone-sensitive prostate cancer (mHSPC), new first-line combination therapies have enhanced overall survival (OS), but clinical outcomes for individual patients vary greatly and are difficult to predict. Peripheral blood circulating tumor cell (CTC) count is the most extensively validated prognostic liquid biomarker in metastatic castration-resistant prostate cancer (mCRPC), and recent studies have suggested that it may also be informative in mHSPC.

**Objective:**

To examine the prognostic value of CTC count in men with mHSPC.

**Design, Setting, and Participants:**

In this prognostic study, peripheral blood was drawn at registration (baseline) and at progression to mCRPC in the S1216 study (March 1, 2013, to July 15, 2017), a phase 3, prospective, randomized clinical trial in men with mHSPC. The CTCs were enumerated using a US Food and Drug Administration–cleared isolation platform. Counts were categorized as 0, 1 to 4, or 5 or more CTCs per 7.5 mL based on the prognostic value of these cut points in prior studies. The data analysis was performed between October 28, 2022, and June 15, 2023.

**Exposure:**

Metastatic hormone-sensitive prostate cancer.

**Main Outcomes and Measures:**

Circulating tumor cell count was evaluated for an association with 3 prespecified trial end points: OS, progression-free survival, and 7-month prostate-specific antigen, after adjusting for other baseline covariates using proportional hazards and logistic regression models.

**Results:**

Of 1313 S1216 participants (median [IQR] age, 68 [44-92] years), evaluable samples from 503 (median [IQR] age, 69 [46-90] years) with newly diagnosed mHSPC were collected at baseline, and 93 samples were collected at progression. Baseline counts were 5 or more CTCs per 7.5 mL in 60 samples (11.9%), 1 to 4 CTCs per 7.5 mL in 107 samples (21.3%), and 0 CTCs per 7.5 mL in 336 samples (66.8%). Median OS for men with 5 or more CTCs per 7.5 mL was 27.9 months (95% CI, 24.1-31.2 months) compared with 56.2 months (95% CI, 45.7-69.8 months) for men with 1 to 4 CTCs per 7.5 mL and not reached at 78.0 months follow-up for men with 0 CTCs per 7.5 mL. After adjusting for baseline clinical covariates, men with 5 or more CTCs per 7.5 mL at baseline had a significantly higher hazard of death (hazard ratio, 3.22; 95% CI, 2.22-4.68) and disease progression (hazard ratio, 2.46; 95% CI, 1.76-3.43) and a lower likelihood of prostate-specific antigen complete response (odds ratio, 0.26; 95% CI, 0.12-0.54) compared with men with 0 CTCs per 7.5 mL at baseline. Adding baseline CTC count to other known prognostic factors (covariates only: area under the curve, 0.73; 95% CI, 0.67-0.79) resulted in an increased prognostic value for 3-year survival (area under the curve, 0.79; 95% CI, 0.73-0.84).

**Conclusions and Relevance:**

In this prognostic study, the findings validate CTC count as a prognostic biomarker that improved upon existing prognostic factors and estimated vastly divergent survival outcomes regardless of subsequent lines of therapy. As such, baseline CTC count in mHSPC may serve as a valuable noninvasive biomarker to identify men likely to have poor survival who may benefit from clinical trials of intensified or novel regimens.

## Key Points

**Question:**

Is circulating tumor cell (CTC) count at the start of treatment associated with overall survival in metastatic hormone-sensitive prostate cancer (mHSPC)?

**Findings:**

In this prognostic study of 1313 men initiating systemic hormonal therapy for mHSPC participating in the prospective, phase 3 S1216 randomized clinical trial, elevated CTC count at baseline was associated with statistically significantly worse overall survival, progression-free survival, and treatment response.

**Meaning:**

These findings validate CTC count as a prognostic biomarker that improves upon existing prognostic factors and estimates vastly divergent survival outcomes regardless of subsequent lines of therapy.

## Teaser

This prognostic study examines the prognostic value of circulating tumor cell count in patients with metastatic hormone-sensitive prostate cancer (mHSPC).

## Introduction

Metastatic prostate cancer is the second leading cause of cancer mortality in US men,^1^ and the incidence of metastatic disease has been increasing.^2,3^ For men who present with metastatic hormone-sensitive prostate cancer (mHSPC), expected survival has improved due to a growing armamentarium of life-extending therapies. Androgen receptor signaling inhibitors (ARSIs) and chemotherapy have progressively moved into earlier use in mHSPC, where they have demonstrated a survival benefit in combination with androgen suppression.^4,5,6,7,8,9,10,11^ In these seminal studies, treatment intensification and added toxicities were deemed to be justified in patients likely to experience poor outcomes based on tumor histologic grade and radiographic disease burden. However, these definitions varied and were not consistently associated with outcomes, underscoring the need for robust prognostic factors to stratify or assign patients to new combination regimens.

In the past decade, liquid biopsy has emerged as an attractive noninvasive approach to analyzing blood-based biomarkers at multiple time points. Tumor-derived analytes such as circulating tumor cells (CTCs) and circulating tumor DNA hold great promise for early cancer detection and prognosis, personalized treatment selection, and monitoring of disease response.^12,13,14,15,16,17^ Major advances have been made in molecular assays using next-generation sequencing for analysis of variant profiles, transcriptional pathways, and methylation patterns. These assays have generated candidate molecular signatures associated with clinical outcomes in retrospective studies.^18,19,20,21^ However, to date, these approaches have yet to be clinically validated as robust prognostic or predictive biomarkers, and their implementation in clinical settings is the focus of ongoing studies.

Among liquid biopsy analytes, CTC count was the first clinically validated prognostic biomarker. Circulating tumor cells are shed from tumor sites into the bloodstream and can potentially seed new metastases. As such, a higher CTC count generally connotes higher tumor burden and more aggressive disease. In metastatic castration-resistant prostate cancer (mCRPC), CTC enumeration on the US Food and Drug Administration–cleared CellSearch platform (Menarini Silicon Biosystems, Inc) has been extensively clinically validated. Our group and others have shown that high baseline CTC count is a strong predictor of poor overall survival (OS) and disease progression in mCRPC, whereas a decrease in CTC counts after treatment is associated with improved outcomes.^22,23,24,25,26^ In mHSPC, far fewer studies have been conducted, but in 2 small cohorts, our group identified CTC counts of 0, 1 to 4, and 5 or more CTCs per 7.5 mL as cut points associated with clinical outcomes.^27,28^

To formally evaluate CTC count as a biomarker in mHSPC, we integrated CellSearch enumeration into S1216, a prospective, multicenter, phase 3 randomized clinical trial coordinated by SWOG on behalf of the National Cancer Institute–sponsored National Clinical Trials Network in collaboration with Alliance, ECOG-ACRIN Cancer Research Group, and NRG Oncology. In S1216, men with mHSPC were randomized to androgen deprivation therapy (ADT) combined with either orteronel (a CYP17 inhibitor) or bicalutamide. In a preliminary analysis, our group reported that our previously identified cut points of 0, 1 to 4, and 5 or more CTCs per 7.5 mL at baseline were prognostic of 7-month prostate-specific antigen (PSA) response and progression-free survival (PFS), the first such evidence from a large, prospective, multicenter trial.^29^ However, at that time, the trial’s final end point data were not yet mature, so we could not assess whether CTC count was prognostic of OS, an important question given the multiple subsequent lines of therapy currently available to treat disease progression. Recently, the final outcomes data of S1216 were published.^30^ While orteronel was associated with an increased PSA response and PFS compared with combined androgen blockade, it did not ultimately result in improved OS, further underscoring the need for baseline biomarkers that can predict long-term outcomes despite multiple subsequent lines of therapy.^30^ The aim of the current study is to report the prognostic value of baseline CTC count for OS in this cohort of men initiating therapy for mHSPC.

## Methods

### Clinical Cohort

This prognostic study followed the Transparent Reporting of a Multivariable Prediction Model for Individual Prognosis or Diagnosis (TRIPOD) reporting guideline.^31^ The clinical trial and correlative study protocols, including the current prognostic study, were approved by the National Cancer Institute’s Cancer Therapy Evaluation Program and Central Institutional Review Board and by the institutional review boards of all participating centers. Written informed consent was obtained from all participants in the clinical trial and correlative study, and the studies were conducted in accordance with ethical guidelines outlined in the US Common Rule.^32^

The S1216 trial (the trial protocol is published in Agarwal et al^30^) included men with newly diagnosed mHSPC initiating therapy. From March 1, 2013, to July 15, 2017, trial participants were randomized 1:1 to combined androgen deprivation (ADT plus bicalutamide) or to ADT plus orteronel (a CYP17 inhibitor). Androgen deprivation therapy was administered using a luteinizing hormone–releasing hormone agonist, while bicalutamide was administered orally at a dose of 50 mg once daily. Orteronel was administered orally at a dose of 300 mg twice daily. Treatment allocation was balanced by 3 stratification factors: disease severity limited to vertebrae and/or pelvic bones and/or lymph nodes (minimal) vs extending to other areas (extensive), ADT initiated within the month prior to enrollment or after enrollment, and Zubrod performance status of 0 to 1 vs 2 to 3. The trial aimed to accrue 1186 eligible participants, with OS as the primary end point. Secondary end points included PFS and PSA levels at 7 months (28 weeks after randomization) dropping to 0.2 ng/mL or less; 0.2 to 4.0 ng/mL; or more than 4.0 ng/mL, which has been shown to be an intermediate end point for OS.^33,34^

### Sample Collection and Processing

S1216 incorporated liquid biopsy translational studies as a Cancer Therapy Evaluation Program and Central Institutional Review Board–approved amendment to the protocol. At baseline and at the time of progression to mCRPC, a 7.5-mL CellSave preservative (Menarini Silicon Biosystems, Inc) blood sample was collected by standard peripheral venipuncture< with written informed consent, from November 18, 2014, to January 12, 2021. Immediately after collection, the tube was placed in a prelabeled mailing kit provided by the investigators and shipped overnight at room temperature to the Goldkorn Laboratory and Liquid Biopsy Core at the USC Norris Comprehensive Cancer Center. Upon arrival, blood samples were processed on the CellSearch platform by a certified technician (T.X.) following the manufacturer’s instructions as described previously.^29^ Briefly, this process uses immunomagnetic beads to target epithelial cell adhesion molecules on the cell surface, which enrich CTCs. The CTCs are then identified through immunofluorescent staining of cytokeratins (CKs) and leukocyte antigen CD45, and nuclear staining with 4′,6-diamidino-2-phenylindole (DAPI). Candidate CTCs that are CK^+^CD45^−^DAPI^+^ are enumerated through a semiautomated imaging algorithm, which is verified by an operator.

### Statistical Analysis

The CellSearch platform was used to process and enumerate candidate CTCs in baseline blood samples drawn from study participants without access to their clinical data. Data on CTC counts were then submitted to the SWOG Statistical Center for correlation with baseline disease and patient characteristics and 3 clinical outcomes: 7-month PSA, PFS, and OS. At baseline, CTC cut points of 0 vs 1 to 4 vs 5 or more cells per 7.5 mL were used based on prior associations identified in mHSPC in our group’s prior studies.^27,29^ At progression, the standard CellSearch cut points of less than 5 CTCs per 7.5 mL and 5 or more CTCs per 7.5 mL were applied.^23,24,35^ The Kruskal-Wallis test and Cochran-Mantel-Haenszel test were used to evaluate correlations of CTC counts with continuous and categorical baseline factors, respectively. The statistical analyses reported in this study were performed between October 28, 2022, and June 15, 2023, using SAS, version 9.4 software (SAS Institute Inc).

The 7-month PSA response outcomes in this study were defined as follows: a complete response (CR) for PSA of 0.2 ng/mL or less, partial response (PR) for PSA between 0.2 and 4.0 ng/mL, and no response for PSA greater than 4.0 ng/mL. Patients who did not have PSA reported for this time point were categorized as nonresponders. Progression-free survival was defined as the duration from randomization to the first occurrence of progression, symptomatic deterioration, or death from disease. Progression was determined by a 25% relative and 2 ng/mL absolute increase in PSA from nadir, a 20% increase in the sum of diameters of soft tissue lesions on computed tomography or magnetic resonance imaging (per Response Evaluation Criteria in Solid Tumors, version 1.1 guidelines^36^), or 2 or more new bone lesions on bone scan. Overall survival was defined as the period from randomization to death from any cause.

The primary end point of OS and secondary end points of 7-month PSA and PFS were assessed across both treatment arms and adjusted for 2 prespecified stratification factors: disease severity (extensive vs minimal) and ADT status prior to enrollment (initiated or not). The end points were also adjusted for all previously identified risk factors that our group collected, including age, self-reported race (Asian, Black, White, multiracial or other [Native American, Pacific Islander], unknown), bone metastases, bone pain, and levels of alkaline phosphatase, hemoglobin, and baseline PSA. Worse performance status was not included as an adjustment factor in the analyses because it was strongly correlated with disease extent. Gleason score also was not used because it was not associated with PFS or OS, and it was missing for a substantial number of patients who were diagnosed and enrolled based on metastasis biopsy. Treatment allocation was dynamically balanced for these factors, which were outlined in the clinical protocol.^30^ Survival was defined from date of randomization to death from any cause, and a PFS event was the first incidence of progression or death. Censoring was at the last contact date.

For the association of baseline CTC count with 7-month PSA response, a polytomous logistic regression model was used, and multinomial logistic regression was used to assess CTC count (categories of 0, 1-4, ≥5 CTCs per 7.5 mL) as a potential prognostic factor of 7-month PSA CR and PR. For the association of baseline CTC counts with PFS and OS, a Cox proportional hazards model was applied with covariate adjustment for the previously specified factors. All *P* values reported are 2-sided and considered significant at the .05 level. A time-dependent model evaluating the interaction of survival time with disease severity, treatment arm, and CTC category was fit, and the χ^2^ test with 3 *df* was evaluated to assess the proportional hazards assumption. The association of CTC count at progression to mCRPC with subsequent OS was evaluated using CTC categories of less than 5 vs 5 or more cells per 7.5 mL, the most extensively validated cut point in prior studies.^22,23,24,25,37^ For the association of progression CTC count with OS, a Cox proportional hazards model was used, adjusting for treatment arm and disease severity at baseline, and then a time-dependent covariate for CTC (dichotomous category) was used to evaluate the association of CTCs at the time of progression with survival, defined as starting at randomization.

Receiver operating characteristic curves were generated by fitting a logistic regression model to survival status at 3 years with the previously listed set of covariates in the model, and the area under the curve (AUC) was estimated along with the 95% CI. A second model was then fit, adding 2 indicators for the CTC baseline category, and the AUC was calculated again. Of the 503 patients with baseline CTCs, 23 (4.6%) withdrew consent or were lost to follow-up prior to year 3 and are excluded from the AUC analysis. However, these participants are included in the Cox OS analysis, censoring on the last day of follow-up.

## Results

### Study Cohort

**Figure 1.**
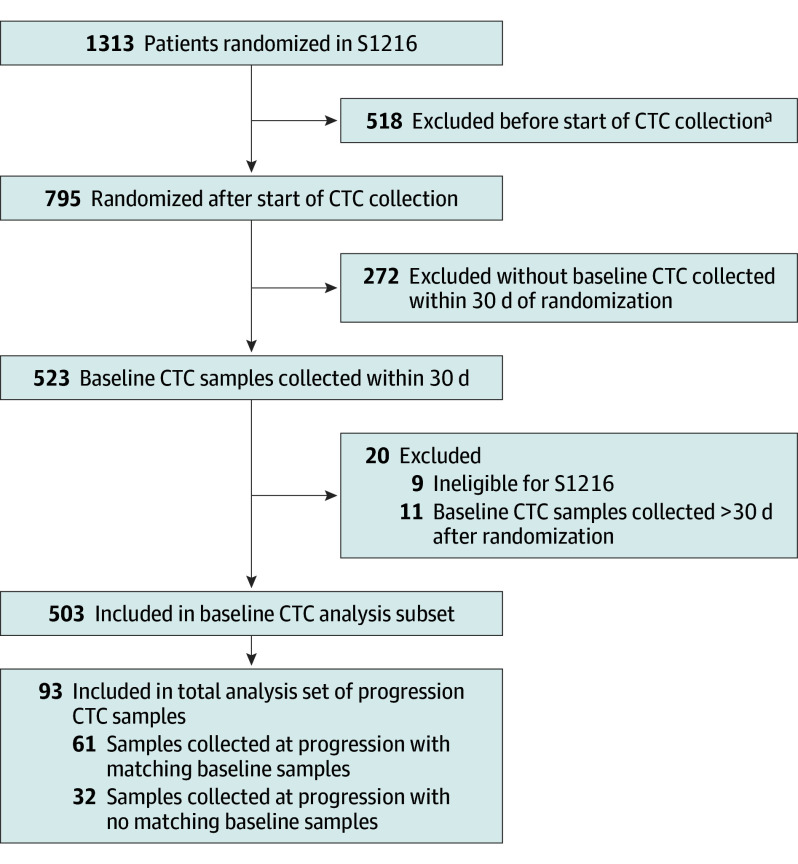
Flow Diagram for Baseline and Progression Circulating Tumor Cell (CTC) Analysis in S1216 ^a^The CTC collection was added in a protocol amendment and implemented by sites by October 30, 2014.

S1216 recruited 1313 men with newly diagnosed mHSPC between 2013 and 2017 (median [IQR] age, 68 [44-92] years; 23 identifying as Asian [1.8%], 142 as Black [10.8%], 1103 as White [84.0%], 8 as multiracial or other [0.6%], and 37 as unknown race [2.8%]). As depicted in the flow diagram in Figure 1, of the 795 patients randomized after activation of the CTC collection amendment, 523 (65.8%) submitted baseline CTC samples, of whom 503 were eligible for baseline CTC analysis. A total of 373 samples (74.2%) were collected prior to randomization or within 7 days after randomization (477 samples [94.8%] within 14 days). Among these 503 patients with baseline samples, 61 (12.1%) also had matching samples collected at progression. An additional 32 samples were collected at progression from patients who had not submitted baseline samples, for a total of 93 CTC progression samples. Distribution of baseline characteristics for the subset of patients with CTC samples was representative of the overall trial (Table 1). Of the baseline characteristics, alkaline phosphatase (median, 83.0 [IQR, 66.0-131.0]; *P* < .001), hemoglobin (median, 14.3 [IQR, 13.1-15.1]; *P* = .004), PSA (median, 26 [IQR, 9-90]; *P* < .001), bone pain (present, 112 [22.3%]; *P* < .001), bone metastases (present, 366 [72.8%]; *P* < .001), and disease severity (extensive, 227 [45.1%]; minimal, 276 [54.9%]; *P* < .001) were all significantly correlated with baseline CTC count (Table 2).

**Table 1.** Baseline Covariates for All Patients and the Subset With Baseline CTC Samples Included in Analyses

Covariate	No. (%)	
	All S1216 patients (N = 1313)	Patients with baseline CTC samples (n = 503)
Age, median (IQR), y	68 (44-92)	69 (46-90)
Race		
Asian	23 (1.8)	9 (1.8)
Black	142 (10.8)	43 (8.5)
White	1103 (84.0)	435 (86.5)
Multiracial or other^a^	8 (0.6)	1 (0.2)
Unknown	37 (2.8)	14 (2.8)
ALP, median (IQR), U/L	88 (68-153)	83 (66-131)
Hb, median (IQR), g/dL	14.2 (13.1-15.1)	14.3 (13.1-15.1)
PSA, median (IQR), ng/mL	30 (10-107)	26 (9-90)
Bone pain	308 (23.5)	112 (22.3)
Bone metastases	980 (74.6)	366 (72.8)
Disease severity		
Extensive	642 (48.9)	227 (45.1)
Minimal	671 (51.1)	276 (54.9)

Abbreviations: ALP, alkaline phosphatase; CTC, circulating tumor cell; Hb, hemoglobin; PSA, prostate-specific antigen.

^a^ Other races included Native American and Pacific Islander.

**Table 2.** Covariate Distribution by Baseline CTC Category and Association Between Baseline and Progression CTC Categories (<5, ≥5 Cells per 7.5 mL) Among Patients Who Submitted Samples at Both Time Points

	Baseline CTC categories, cells/7.5 mL			
	0	1-4	≥5	*P* value^a^
No. of patients	336	107	60	NA
Age, median (IQR), y	68.6 (62.9-74.0)	69.9 (62.5-76.4)	68.2 (63.3-75.1)	.53
White race, No. (%)	294 (68)	89 (20)	52 (12)	.54
ALP, median (IQR), U/L	79 (62-109)	89 (72-173)	121 (94-307)	<.001
Hb, median (IQR), g/dL	14.3 (13.4-15.3)	14.1 (12.9-15.1)	13.9 (12.3-14.8)	.004
PSA, median (IQR), ng/mL	20 (8-64)	37 (12-114)	93 (18-277)	<.001
Bone pain, No. (row %)				
Yes	63 (56)	23 (21)	26 (23)	<.001
No	273 (70)	84 (21)	34 (9)	
Bone metastases, No. (row %)				
Yes	224 (61)	87 (24)	55 (15)	<.001
No	112 (82)	20 (14)	5 (4)	
Disease severity, No. (row %)				
Extensive	125 (55)	59 (26)	43 (19)	<.001
Minimal	211 (77)	48 (17)	17 (6)	
Progression CTC count per 7.5 mL				
No. of patients	34	17	10	
<5	27 (79)	12 (71)	3 (30)	.01^b^
≥5	7 (21)	5 (29)	7 (70)	

Abbreviations: ALP, alkaline phosphatase; CTC, circulating tumor cell; Hb, hemoglobin; NA, not applicable; PSA, prostate-specific antigen.

^a^ Kruskal-Wallis test for continuous covariates and Cochran-Mantel-Haenszel test for categorical covariates.

^b^ Spearman correlation.

### Correlation of Baseline CTC Count With OS, PSA Response, and PFS

**Figure 2.**
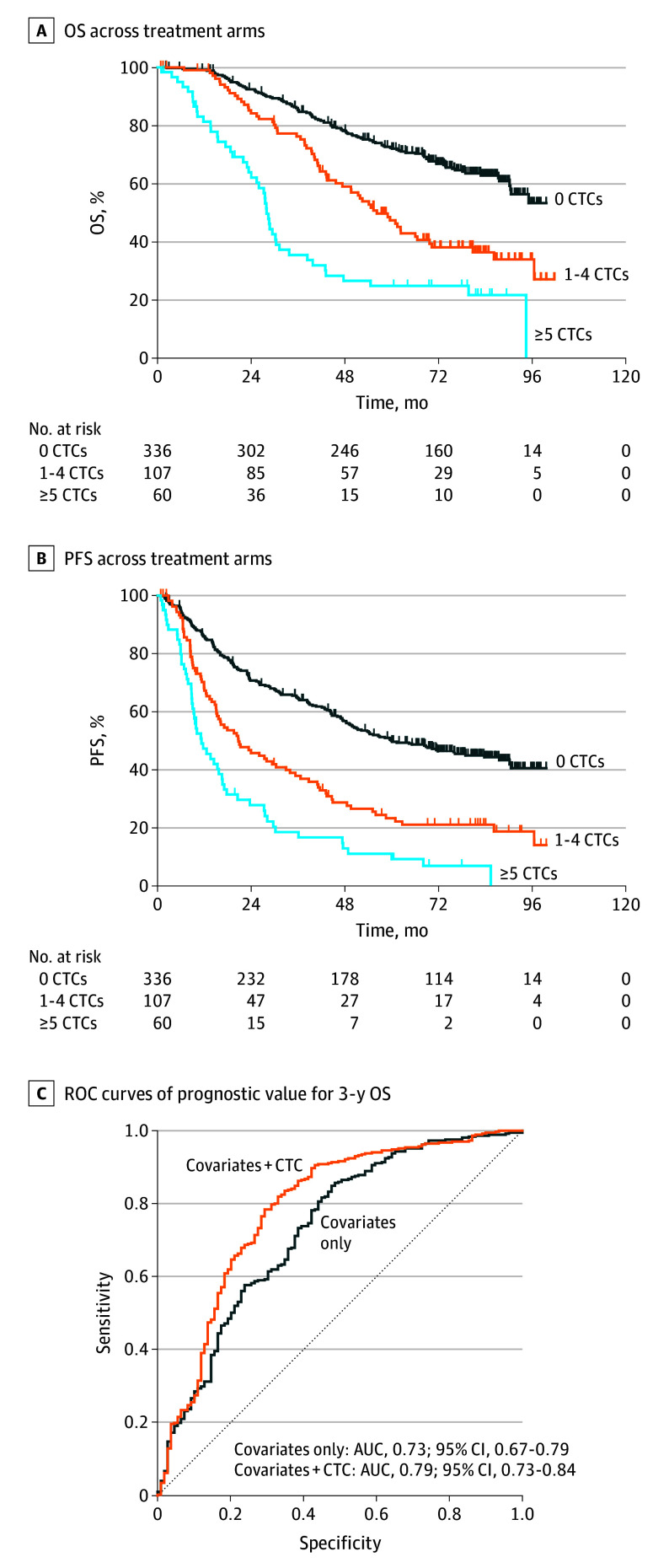
Baseline Circulating Tumor Cell (CTC) Count per 7.5 mL and Overall Survival (OS) and Progression-Free Survival (PFS) Tic marks indicate censored data. AUC indicates area under the curve; ROC, receiver operating characteristic.

Baseline CTC count in mHSPC was 5 or more cells per 7.5 mL in 60 samples (11.9%), 1 to 4 cells per 7.5 mL in 107 samples (21.3%), and 0 cells per 7.5 mL in 336 samples (66.8%). The median OS for patients with 5 or more CTCs per 7.5 mL was 27.9 months (95% CI, 24.1-31.2 months) compared with 56.2 months (95% CI, 45.7-69.8 months) for patients with 1 to 4 CTCs per 7.5 mL and not reached for men with 0 CTCs per 7.5 mL at time of reporting after a median follow-up period of 78.0 months (Figure 2A). Compared with patients with 0 CTCs per 7.5 mL at baseline, the hazard of death was higher in men with 1 to 4 CTCs per 7.5 mL (hazard ratio [HR], 1.88; 95% CI, 1.36-2.61; *P* < .001) and in those with 5 or more CTCs per 7.5 mL (HR, 3.22; 95% CI, 2.22-4.68; *P* < .001) after adjusting for other baseline covariates (Table 3). Similar associations were found in the individual treatment arms (eFigure 1A and B in Supplement 1).

**Table 3.** Multivariable Proportional Hazards Modeling of Overall Survival and Progression-Free Survival (n = 503)

Covariate	Progression-free survival		Overall survival	
	HR (95% CI)	*P* value	HR (95% CI)	*P* value
Extensive disease	1.64 (1.28-2.10)	<.001	1.59 (1.17-2.16)	.003
Treatment arm: orteronel vs CAD	0.62 (0.49-0.79)	<.001	1.02 (0.77-1.35)	.92
Age	1.01 (0.99-1.02)	.21	1.01 (0.99-1.03)	.17
White race	0.92 (0.67-1.27)	.63	0.90 (0.61-1.31)	.58
Hemoglobin	0.95 (0.88-1.02)	.16	0.92 (0.84-1.00)	.06
Alkaline phosphatase	1.00 (1.00-1.00)	.03	1.00 (0.99-1.00)	.35
PSA	1.00 (0.99-1.00)	.33	1.00 (0.99-1.00)	.64
Bone metastasis	1.61 (1.18-2.18)	.002	1.65 (1.10-2.48)	.01
Bone pain	1.18 (0.88-1.58)	.26	1.23 (0.88-1.73)	.22
Baseline CTC count, per 7.5 mL				
≥5	2.46 (1.76-3.43)	<.001	3.22 (2.22-4.68)	<.001
1-4	1.74 (1.32-2.29)	<.001	1.88 (1.36-2.61)	<.001

Abbreviations: CAD, combined androgen deprivation; CTC, circulating tumor cell; HR, hazard ratio; PSA, prostate-specific antigen.

In both the combined androgen deprivation and orteronel treatment arms, PSA CR was observed most frequently in patients with 0 CTCs per 7.5 mL (79 [58.5%] and 137 [68.2%], respectively), whereas no PSA response was most frequently observed in those with 5 or more CTCs per 7.5 mL (16 [50.0%] and 13 [46.4%], respectively) (eFigure 1C in Supplement 1). Based on these distributions, patients with a baseline count of 5 or more CTCs per 7.5 mL were significantly less likely to achieve a PR (odds ratio [OR], 0.45, 95% CI, 0.21-0.96, *P* = .03) or CR (OR, 0.26; 95% CI, 0.12-0.54, *P* < .001) for PSA compared with those with 0 CTCs per 7.5 mL after adjusting for other baseline covariates (eTable 1 in Supplement 1).

Median PFS for patients with 5 CTCs or more per 7.5 mL was 11.3 months (95% CI, 8.8-16.5 months) vs 20.7 months (95% CI, 15.1-32.9 months) and 59.9 months (95% CI, 49.4-83.7 months) for patients with 1 to 4 and 0 CTCs per 7.5 mL, respectively (Figure 2B). Compared with patients with 0 CTCs per 7.5 mL, the likelihood of disease progression was higher in those with 1 to 4 CTCs per 7.5 mL (HR, 1.74; 95% CI, 1.32-2.29; *P* < .001) and with 5 or more CTCs per 7.5 mL (HR, 2.46; 95% CI, 1.76-3.43; *P* < .001) after adjusting for other baseline covariates (Table 3). Similar associations were observed in the individual treatment arms (eFigure 2A and B in Supplement 1). The addition of baseline CTC counts resulted in improved prognostic power over 3 years, with the AUC increasing from 0.73 (95% CI, 0.67-0.79) for covariates only to 0.79 (95% CI, 0.73-0.84) for covariates plus CTCs (Figure 2C).

### Correlation of Progression CTC Count With OS and Baseline CTC Count

Median postprogression OS for patients with 5 or more CTCs per 7.5 mL was 15.4 months (95% CI, 10.3-19.8 months) vs 41.9 months (95% CI, 29.3-50.6 months) for patients with less than 5 CTCs per 7.5 mL. Patients with 5 or more CTCs per 7.5 mL at progression had a higher hazard of death than those with less than 5 CTCs per 7.5 mL (HR, 4.01; 95% CI, 2.21-7.27; *P* < .001) in the time-dependent model, associated with their median survivals of 15.4 and 41.9 months, respectively (eFigure 3 in Supplement 1). Progression CTC counts were not significantly associated with baseline patient characteristics (eTable 2 in Supplement 1). Sixty-one patients had matched baseline and progression CTC counts. Most of the patients with baseline CTCs of 0 and 1 to 4 cells per 7.5 mL (27 of 34 [79%] and 12 of 17 [71%], respectively) also had fewer than 5 CTCs per 7.5 mL at progression, whereas most of the patients with baseline counts of 5 or more CTCs per 7.5 mL (7 of 10 [70%]) also had progression counts of 5 or more CTCs per 7.5 mL. Overall, the matched baseline progression CTC count pairs were highly correlated (Spearman *P* = .01) (Table 2).

## Discussion

In the past decade, therapies approved for mCRPC have been progressively integrated into standard first-line treatment for mHSPC. Upfront intensification using double or even triple therapy comprising ADT plus ARSIs and/or chemotherapies has enhanced OS in mHSPC^4,5,7,8,9,10,11^ but entails additional toxicities. In general, patients expected to derive the greatest benefit from upfront intensification have been those with more aggressive disease. Specifically, the CHAARTED and LATITUDE trials established categorizations of high- vs low-risk and high- vs low-volume disease, respectively.^4,5^ While useful, these definitions offer imperfect and somewhat overlapping classification, as evidenced in the ARASENS trial, wherein survival benefit was not restricted only to patients with high-risk or high-volume disease.^10,38^ Therefore, an accurate noninvasive biomarker to select patients with a poor prognosis at the start of therapy may advance ongoing and future clinical development of mHSPC treatments.

In mCRPC, a variety of blood-based assays have been assessed in recent years as potential biomarkers to guide disease management. As next-generation sequencing technologies improved and became more accessible, various targeted sequencing approaches have been used to gauge the prognostic importance of prostate cancer–relevant molecular alterations. These have included specific genes such as *AR*, *PTEN*, and *PIK3CA* and homologous recombination repair genes (eg, *BRCA1* and *BRCA2*), as well as broader molecular features such as tumor mutation burden and circulating tumor DNA fraction of total cell-free DNA.^16,39,40,41,42^ Most of these assays have focused on cell-free DNA, but some analyzed CTCs, most prominently for the presence of the androgen receptor splice variant 7 associated with resistance to ARSIs.^16,26,43,44,45^ However, with the exception of homologous recombination repair testing for poly (ADP ribose) polymerase inhibition and rarely tumor mutation burden for checkpoint inhibitors, the majority of liquid biomarkers have not been adopted widely for clinical use. Circulating tumor cell count on the Food and Drug Administration–cleared CellSearch platform is the most extensively validated liquid biomarker for mCRPC, but its use has been limited in this advanced disease state because patients ultimately experience progression through most therapies regardless of their prognosis.

To date, few candidate biomarkers assessed in mCRPC have been formally evaluated in mHSPC. Yet, management of this earlier disease state arguably has more to gain from informative biomarkers as a large majority of men diagnosed with mHSPC experience excellent performance status and a life expectancy measured in years. In these men, selection from a host of upfront therapy combinations—each attended by different risk-to-benefit profiles—would be greatly facilitated by validated outcome predictors. Men predicted to have a poor prognosis could be selected for more intensive or investigational treatments, whereas those with good prognosis, especially if frail or older, could be treated less aggressively. Studies are now starting to address this question using baseline blood-based assays, as done in our group’s recent study of markers of bone turnover in mHSPC.^46^ By that same rationale, we also assessed in the current study the value of baseline CTC count.

In this prognostic study, our results in S1216 constitute, to our knowledge, the largest reported analysis of CTC count as an integrated biomarker in a prospective, phase 3, randomized clinical trial for mHSPC. We found that CTC counts of 0, 1 to 4, and 5 or more cells per 7.5 mL at baseline was associated with 7-month PSA and PFS in the pooled cohort and in each treatment arm, confirming prior reports from our group’s preliminary S1216 analysis and independently validating those cut points from prior smaller studies.^27,28,29^ Moreover, the final reporting of S1216 has now enabled analysis of CTC count as a prognostic factor of OS: Whereas median OS was not reached in men with 0 CTCs per 7.5 mL at baseline, it was 56.2 months in men with 1 to 4 CTCs per 7.5 mL and only 27.9 months in men with 5 or more CTCs per 7.5 mL. Thus, despite multiple lines of subsequent therapy for mCRPC, men with elevated CTCs at the outset of therapy for mHSPC had a 2-fold to 3-fold higher hazard of death than men with no detectable CTCs after adjusting for all relevant covariates. Indeed, baseline CTC count was significantly more prognostic (ie, higher HRs [Table 3]) of PFS and OS than any other clinical variables, and adding CTC count to other baseline covariates improved estimates of 3-year OS (ie, higher AUC [Figure 2C]).

A consistent trend throughout the disease course was also observed in the small subset of 61 patients with matching CTC counts at baseline and progression. In this subgroup, high or low CTC count at presentation with mHSPC was highly correlated with high or low CTC count at progression to mCRPC. This finding suggests that despite the well-documented phenomenon of treatment-emergent molecular alterations and resistant clones, underlying aggressive disease phenotypes associated with high CTC count are present both before and after exposure to treatment selection pressures. In contrast, other prognostic clinical variables that were associated with CTC counts at baseline were no longer associated with CTC count at progression (eTable 2 in Supplement 1).

### Limitations

This study has several limitations. Prospective CTC collection was added as a prespecified correlative end point via a protocol amendment after study accrual had begun, so baseline CTC analysis could not be conducted on the entire S1216 cohort. However, as shown in Table 1, the CTC analysis cohort was representative of the overall cohort across all demographic and disease-relevant covariates. Also, the study drug orteronel, a CYP17 inhibitor like abiraterone, exhibited PFS benefit and PSA responses similar to what has been seen with currently approved *AR* pathway inhibitors, but the trial did not meet its OS end point for regulatory approval. This shortfall was attributed to the unexpectedly long survival in the standard treatment arm, as patients who experienced progression to mCRPC were treated with new life-extending therapies.^30^ The association of baseline CTC count with OS across arms despite these many lines of therapy highlights the potential prognostic value of this biomarker. In addition, the S1216 protocol collected the date of metastatic disease diagnosis but did not record information about initial prostate cancer diagnosis or whether a prostatectomy was performed, so it was not possible to assess whether study participants initially presented with metachronous vs synchronous disease. Finally, our results apply to men who have not started treatment or have received up to 30 days of ADT. We adopted this commonly used 30-day window due to the logistical practicalities posed by registering patients, initiating treatment, and collecting correlative laboratory results, all within the shortest possible time span. That 94.8% of baseline CTC samples were collected within 14 days and 74.2% were collected within 7 days of registration is reassuring.

## Conclusions

Collectively, the results of this prognostic study show that baseline elevated CTC count was associated with poor response, rapid progression, and poor survival, reflecting innate aggressive phenotypes that remain consistent throughout the disease course and after subsequent lines of therapy. Given these characteristics, baseline CTC count may be used to facilitate clinical development of new, more effective treatments. Specifically, in men with newly diagnosed mHSPC, of whom two-thirds generally have good performance status and years of life expectancy,^30^ baseline CTC count may identify the one-third of men with more aggressive disease who are likely to experience worse outcomes. This prognostic ability may be of particular benefit in the slate of new clinical trials being launched to test standard mHSPC treatment vs intensified triple therapy (ADT, ARSI, chemotherapy) or other novel combinations. In this new generation of trials, elevated CTC count may serve as a valuable baseline biomarker to enrich the study cohorts for men most likely to benefit from these more aggressive therapeutic strategies.

## Notes

Supplement 1. **eTable 1.** Odds Ratios for Clinical Covariates and Baseline CTC Count in a Multivariable Polytomous Logistic Regression Model Predicting PSA Response **eTable 2.** Analysis of Baseline Covariate Distribution by Progression CTC Categories **eFigure 1.** Baseline CTC Categories (0, 1-4, ≥5 CTCs), PSA Response, and Overall Survival by Treatment Arm **eFigure 2.** Baseline CTC Categories (0, 1-4, ≥5 CTCs) and Progression-Free Survival by Treatment Arm **eFigure 3.** Progression CTC Count (<5 CTCs, ≥5 CTCs) and Overall Survival (n = 93)

Supplement 2.
Data Sharing Statement
