# Supplementary material for: Circulating Tumor Cell Count and Overall Survival in Patients With Metastatic Hormone-Sensitive Prostate Cancer
Source: JAMA Netw Open. 2024 Oct 7;7(10):e2437871. doi: 10.1001/jamanetworkopen.2024.37871 (PMC11581504; doi:10.1001/jamanetworkopen.2024.37871)
Supplement: Supplement 1. — eTable 1. Odds Ratios for Clinical Covariates and Baseline CTC Count in a Multivariable Polytomous Logistic Regression Model Predicting PSA Response eTable 2. Analysis of Baseline Covariate Distribution by Progression CTC Categories eFigure 1. Baseline CTC Categories (0, 1-4, ≥5 CTCs), PSA Response, and Overall Survival by Treatment Arm eFigure 2. Baseline CTC Categories (0, 1-4, ≥5 CTCs) and Progression-Free Survival by Treatment Arm eFigure 3. Progression CTC Count (<5 CTCs, ≥5 CTCs) and Overall Survival (n = 93) [file jamanetwopen-e2437871-s001.pdf]

Supplemental Online Content

Goldkorn A, Tangen C, Plets M, et al. Circulating tumor cell count and overall survival in patients with metastatic hormone-sensitive prostate cancer. *JAMA Netw Open*. 2024;7(10):e2437871. doi:10.1001/jamanetworkopen.2024.37871

eTable 1. Odds Ratios for Clinical Covariates and Baseline CTC Count in a Multivariable Polytomous Logistic Regression Model Predicting PSA Response

eTable 2. Analysis of Baseline Covariate Distribution by Progression CTC Categories

eFigure 1. Baseline CTC Categories (0, 1-4, ≥5 CTCs), PSA Response, and Overall Survival by Treatment Arm

eFigure 2. Baseline CTC Categories (0, 1-4, ≥5 CTCs) and Progression-Free Survival by Treatment Arm

eFigure 3. Progression CTC Count (<5 CTCs, ≥5 CTCs) and Overall Survival (n = 93)

This supplemental material has been provided by the authors to give readers additional information about their work.

© 2024 Goldkorn A et al. *JAMA Network Open.*

eTable 1. Odds Ratios for Clinical Covariates and Baseline CTC Count in a Multivariable Polytomous Logistic Regression Model Predicting PSA Response

	No Response (N=111)	Partial Response (N=109)	Complete Response (N=283)
**Baseline CTC category**			
**0**	Ref	Ref	Ref
**1-4**	Ref	0.63 (0.32, 1.24) p=0.18	0.58 (0.32, 1.05) p=0.07
**5+**	Ref	0.45 (0.21, 0.96) p=0.03	0.26 (0.12, 0.54) p<0.001
**Treatment arm: Orteronel vs CAD**	Ref	0.55 (0.31, 0.97) p=0.04	1.28 (0.78, 2.12) p=0.33
**Disease Severity: Extensive vs Minimal**	Ref	0.88 (0.48, 1.63) p=0.68	0.57 (0.33, 0.98) p=0.04
**Age (years)**	Ref	0.99 (0.95, 1.02) p=0.46	0.98 (0.95, 1.01) p=0.28
**Race: White vs Non-white**	Ref	1.37 (0.62, 3.01) p=0.44	1.19 (0.59, 2.39) p=0.63
**Alkphos (U/L)**	Ref	1.00 (1.00, 1.00) p=0.54	1.00 (0.99, 1.00) p=0.96
**Hgb (g/dL)**	Ref	1.33, (1.13, 1.56) p<0.001	1.32 (1.13, 1.53) p<0.001
**Baseline PSA* (ng/mL)**	Ref	1.00 (1.00, 1.01) p=0.21	0.99 (0.98, 1.00) p=0.02
**Bone Mets: Yes vs No**		1.45 (0.64, 3.30) p=0.38	0.56 (0.28, 1.09) p=0.09
**Bone Pain: Yes vs No**	Ref	0.60 (0.30, 1.20) p=0.15	0.64 (0.35, 1.17) p=0.15
Treatment x Baseline CTC interaction not significant (p=0.15, 4 df residual chi-sq test)			

© 2024 Goldkorn A et al. *JAMA Network Open.*

eTable 2. Analysis of Baseline Covariate Distribution by Progression CTC Categories

	Progression CTC categories (N=93)**		
	<5 (N=68)	≥5 (N=25)	pval*
**Age (median yrs)**	68	66	0.79
**Race: white (N, %)**	58 (85%)	23 (92%)	0.40
**Alk Phos (median U/L)**	94	147	0.06
**Hgb (median g/dL)**	14.0	13.6	0.38
**PSA (median ng/mL)**	58.7	57.2	0.87
**Bone pain (N, %)**			
Yes	16 (76%)	5 (24%)	0.72
No	52 (72%)	20 (28%)	
**Bone mets (N, %)**			
Yes	49 (69%)	22 (31%)	0.11
No	19 (86%)	3 (14%)	
**Disease severity (N, %)**			
Extensive	37 (69%)	17 (31%)	0.24
Minimal	31 (79%)	8 (21%)	

*Kruskal-Wallis test for continuous covariates, Cochran-Mantel-Haenszel test for categorical covariates
** N=32 submitted progression CTC sample but no baseline CTC sample; N=61 submitted both baseline and progression CTC samples

© 2024 Goldkorn A et al. *JAMA Network Open.*

eFigure 1. Baseline CTC Categories (0, 1-4, ≥5 CTCs), PSA Response, and Overall Survival by Treatment Arm

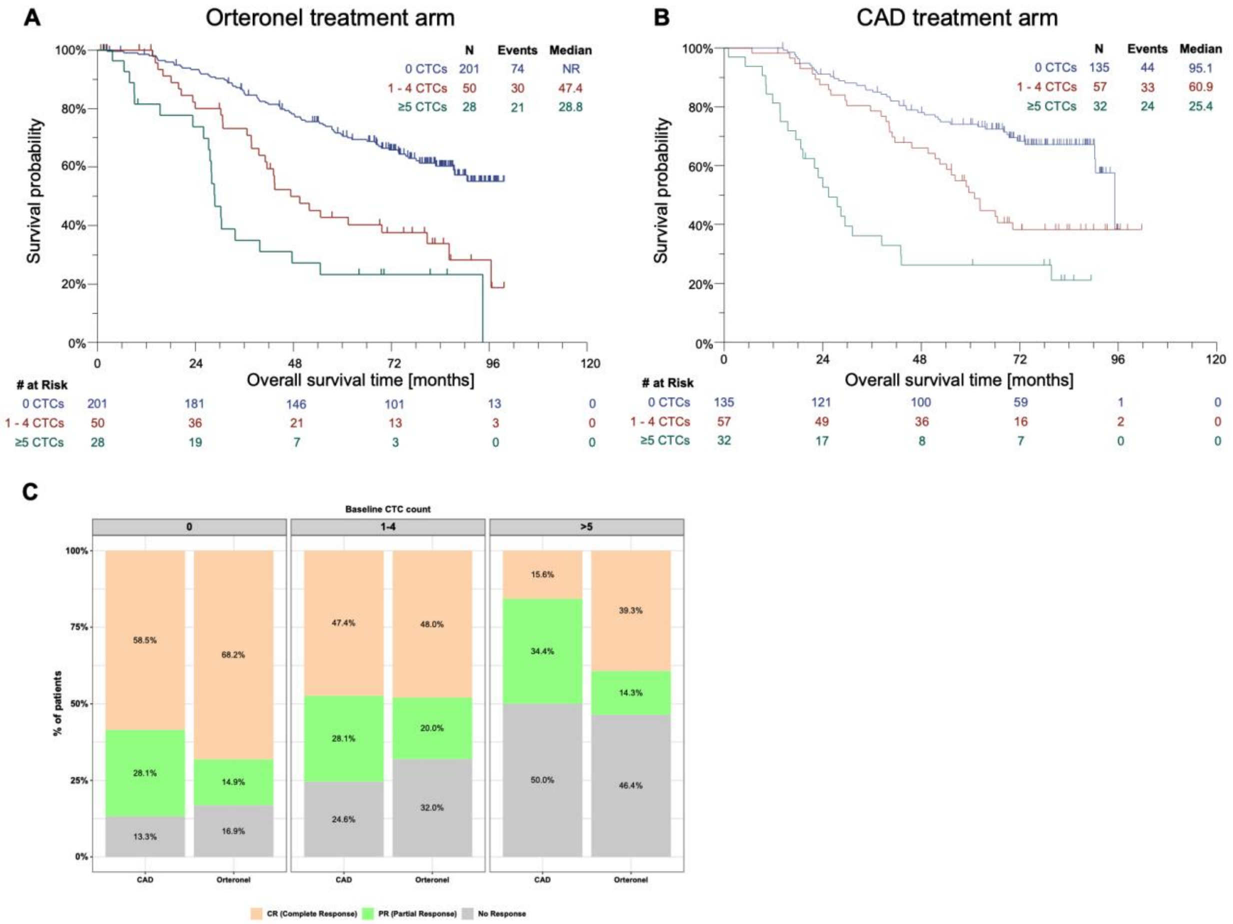

(A) Orteronel treatment arm of patients receiving Androgen deprivation therapy (ADT) and Orteronel (CYP17 inhibitor)) or (B) CAD treatment arm (Combined Androgen Deprivation) of patients receiving ADT and bicalutamide. (C) Association of baseline CTC count categories (0 CTCs, 1-4 CTCs, ≥5 CTCs) with 7-month PSA response (No response, Partial Response, Complete Response) by treatment arms.

© 2024 Goldkorn A et al. *JAMA Network Open.*

eFigure 2. Baseline CTC Categories (0, 1-4, ≥5 CTCs) and Progression-Free Survival by Treatment Arm

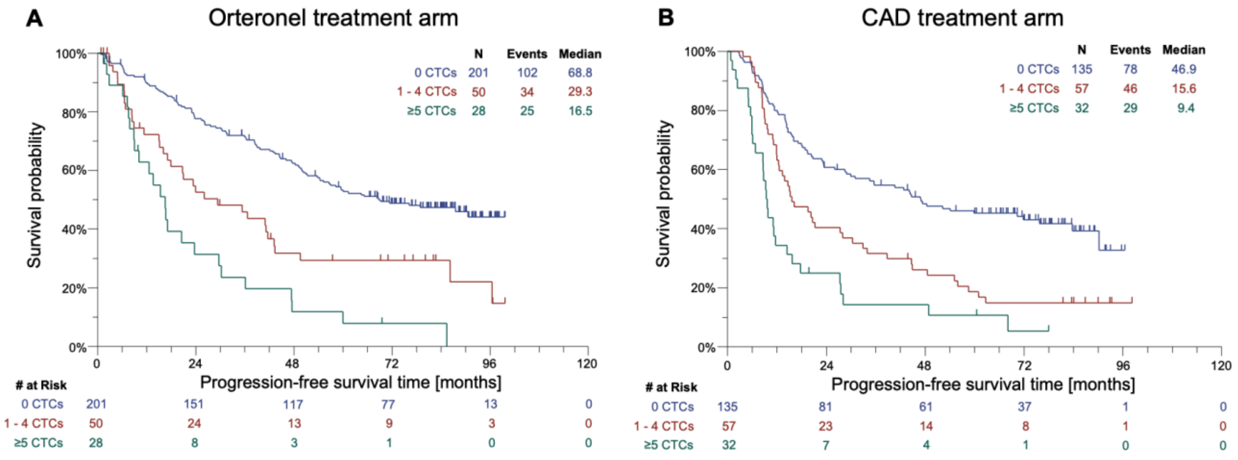

(A) Patients randomized to receive androgen deprivation therapy (ADT) and Orteronel or (B) Patients randomized to receive ADT and bicalutamide. Time (months) is defined from the date of treatment start to PFS event.

© 2024 Goldkorn A et al. *JAMA Network Open.*

eFigure 3. Progression CTC Count (<5 CTCs, ≥5 CTCs) and Overall Survival (n = 93)

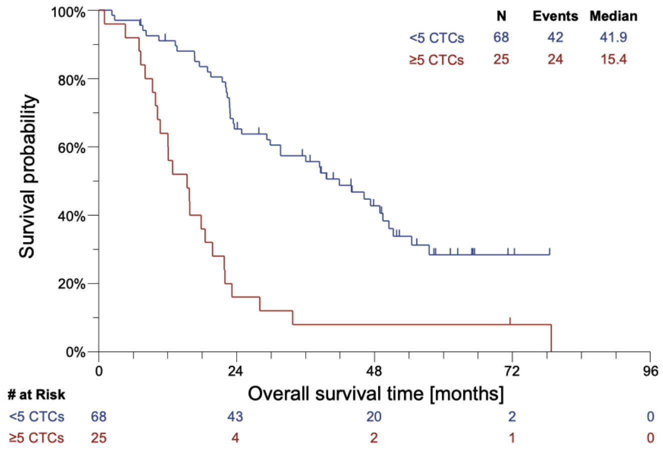

Kaplan-Meier curves by progression CTC categories (<5 and ≥5 CTCs), pooled across treatment arms.

© 2024 Goldkorn A et al. *JAMA Network Open.*
