# Supplementary material for: Circulating Tumor Cell Count and Overall Survival in Patients With Metastatic Hormone-Sensitive Prostate Cancer
Source: JAMA Netw Open. 2024 Oct 7;7(10):e2437871. doi: 10.1001/jamanetworkopen.2024.37871 (PMC11581504; doi:10.1001/jamanetworkopen.2024.37871)
Supplement: Supplement 2. — Data Sharing Statement [file jamanetwopen-e2437871-s002.pdf]

Data Sharing Statement

Goldkorn. Circulating Tumor Cell Count and Overall Survival in Patients With Metastatic Hormone-Sensitive Prostate Cancer. *JAMA Netw Open*. Published October 07, 2024. doi:10.1001/jamanetworkopen.2024.37871

Data
Data available: Yes
Data types: Deidentified participant data
How to access data: Requests for data from SWOG S1216 will be reviewed and granted in collaboration with the NCI SWOG Statistical Core and trial investigators. Requests can be made directly to the corresponding author, Amir Goldkorn, at agoldkor@med.usc.edu.
When available: With publication

Supporting Documents
Document types: Other (please specify)
Additional Information: S1216 clinical trial protocol.
How to access documents: Requests can be made directly to the corresponding author, Amir Goldkorn, at agoldkor@med.usc.edu.
When available: With publication

Additional Information
Who can access the data: Researchers whose proposed use of the data has been approved.
Types of analyses: Analyses associated with the CTC biomarker outcomes reported in this manuscript.
Mechanisms of data availability: After review of a proposal and with signed data access agreement.
